# Goal-directed therapy based on rScO_2_ monitoring in elderly patients with one-lung ventilation: a randomized trial on perioperative inflammation and postoperative delirium

**DOI:** 10.1186/s13063-022-06654-6

**Published:** 2022-08-19

**Authors:** Jing-yu Wang, Ming Li, Pei Wang, Ping Fang

**Affiliations:** 1 Surgical Anesthesia Center, Li Huili Hospital, Ningbo Medical Center, No.57 Xingning Road, Yinzhou District, Ningbo, 315000 Zhejiang Province China; 2 Surgical Anesthesia Center, The Second Hospital of Haishu District, No.52 Yizhi Middle Road, Shiqi Street, Haishu District, Ningbo, 315000 Zhejiang Province China; 3 Department of Neurology, Li Huili Hospital, Ningbo Medical Center, No.57 Xingning Road, Yinzhou District, Ningbo, 315000 Zhejiang Province China

**Keywords:** Regional saturation of cerebral oxygenation, Postoperative delirium, Inflammatory factors, Goal-directed therapy, Lactate, Agitation during awakening, Awakening time, Randomized controlled trial

## Abstract

**Background:**

The incidence of postoperative delirium (POD) is high in elderly patients with one-lung ventilation, which is mostly related to the impairment of cerebral oxygen supply/demand balance during operation. (Surgical) stress can cause changes to normal physiological function and increase oxygen supply to the brain. When cerebral oxygen supply/demand is unbalanced, other organs may have already suffered from hypoperfusion or even hypoxic damages leading to increased release of inflammatory factors. Regional saturation of cerebral oxygenation (rScO_2_) monitoring can noninvasively monitor the variation of regional cerebral oxygen supply/demand balance in real time, and it has a good correlation with the occurrence of POD. S-100β is one of the markers commonly used to predict and diagnose POD, and lactate is one of the important indicators for the quality of tissue perfusion. The study explores whether the goal-directed therapy based on rScO_2_ monitoring can reduce perioperative inflammatory factor levels and POD incidence in elderly patients with one-lung ventilation and improve tissue perfusion.

**Methods:**

The study is registered on Chinese Clinical Trial Registry (ChiCTR2100054888). A total of 159 patients scheduled for thoracoscopic lobectomy under general anesthesia were divided into the control group (*n* = 81) and the goal-directed therapy group (GDT group, *n* = 78). On the basis of the conventional management in the control group, the GDT group applied goal-directed rScO_2_ monitoring to maintain rScO_2_ at ±20% baseline level during one-lung ventilation. The levels of interleukin-1β, interleukin-6, tumor necrosis factor-α, and lactate; the intensity of postoperative pain; and the incidence of POD before anesthesia (T1), at the end of operation (T2), on day 1 after operation (T3), on day 3 after operation (T4), and on day 7 after operation or before discharge (T5) were compared respectively between the two groups.

**Results:**

The incidence of POD at T3 and the awakening time in the GDT group were lower than those in the control group (*P* < 0.05). During T2 to T4, the levels of inflammatory factors and lactate concentration in the control group were higher than those in the GDT group (*P* < 0.05). During T3 to T4, the levels of C-reactive protein and lactate in the control group were higher than those in the GDT group (*P* < 0.05). During T2 to T3, the levels of S-100β in the control group were higher than those in the GDT group (*P* < 0.05). The levels of inflammatory factors and lactate concentration in both groups during T2 to T4 were higher than those at T1 and T5 (*P* < 0.05), and there was no statistical difference at T1 versus T5 (*P* > 0.05). There was no significant difference in postoperative pain intensity, the incidence of agitation during awakening, and postoperative hospital stays between the two groups.

**Conclusion:**

Goal-directed therapy based on rScO_2_ monitoring can reduce perioperative inflammatory factor levels, postoperative delirium incidence, and postoperative awakening time and improve tissue perfusion in elderly patients with one-lung ventilation.

**Trial registration:**

The Chinese Clinical Trial Registry ChiCTR2100054888. Registered on 28 December 2021

**Supplementary Information:**

The online version contains supplementary material available at 10.1186/s13063-022-06654-6.

## Background

Postoperative delirium (POD) is a kind of acute, reversible, and unexplained encephalopathic syndrome occurring within 1 week after surgery (or before discharge), mainly characterized by neurocognitive dysfunction [1]. It seriously affects the life quality and the recovery of patients after surgery and shares a positive relation to the increase of short- and long-term mortality [2]. During one-lung ventilation (OLV), the incidence of POD can be up to 30~50% [3], due to the impaired oxygen supply/demand balance in the brain of elderly patients as a result of high hypoxemia risk caused by decreased ventilation efficiency, lessened oxygen supply, lateral position and intrapulmonary shunting, etc. [4, 5]. Currently, the pathogenesis of POD is unknown, but research revealed associations with cerebral oxygen supply/demand imbalance and the following increased release of inflammatory factors [6].

Multiple studies showed that interleukin-1β (IL-1β), interleukin-6 (IL-6), tumor necrosis factor-α (TNF-α), and C-reactive protein (CRP) play a crucial role in POD incidence [7–9]. The total weight of the brain merely occupies 2% of the whole body, but its oxygen consumption takes up 20% of the total need of the human body. (Surgical) stress can cause changes to normal physiological function and increase oxygen supply to the brain. When cerebral oxygen supply/demand balance is impaired, other organs may have already suffered from hypoxia-induced injury causing the release of inflammatory factors. In the meantime, the inflammatory factors resulted from hypoxia of peripheral organs can enter the brain through the fragile or damaged blood-brain barrier to accelerate POD in elderly patients [10]. The traditional oxygen saturation measured by pulse oximetry (SpO_2_) monitoring only reflects the blood oxygen concentration of end circulation but not the real-time supply/demand balance of the brain, which is an organ with high oxygen consumption. When peripheral SpO_2_ decreases, damages from hypoxia may already occur in the brain and induce POD [11].

Regional saturation of cerebral oxygenation (rScO_2_) monitoring can noninvasively indicate the variation of regional cerebral oxygen supply/demand in real time, and it has a good correlation with the occurrence of POD [12]. The S-100β is a specific protein produced by the glial cells and it can be released to peripheral blood circulation when POD occurs, which suggests an association between the blood S-100β protein content and the degree of damage to the cerebral glial cells [13]. Glucose is the exclusive energy source of brain cells, and when the oxygen supply/demand is imbalanced, lactate increases due to the anaerobic metabolism.

The study applies a goal-directed therapy based on rScO_2_ monitoring in elderly patients with OLV, in an attempt to explore whether it is superior to conventional management strategy in reducing perioperative levels of inflammatory factors and POD incidence and improving peripheral tissue perfusion, so as to provide a new method for perioperative organ protection.

## Methods

### Study design

A prospective randomized single-blind controlled clinical trial was scheduled for the study. The trial was approved by the Ethics Committee of Ningbo Medical Center Lihuili Hospital (KY2019PJ001) and was registered on Chinese Clinical Trial Registry (ChiCTR) (No: ChiCTR2100054888). Informed consent was signed after patients and their immediate families were informed of the benefits and risks associated with this study. All relevant original data of this study are reserved in our hospital.

### Participants

**Fig. 1:**
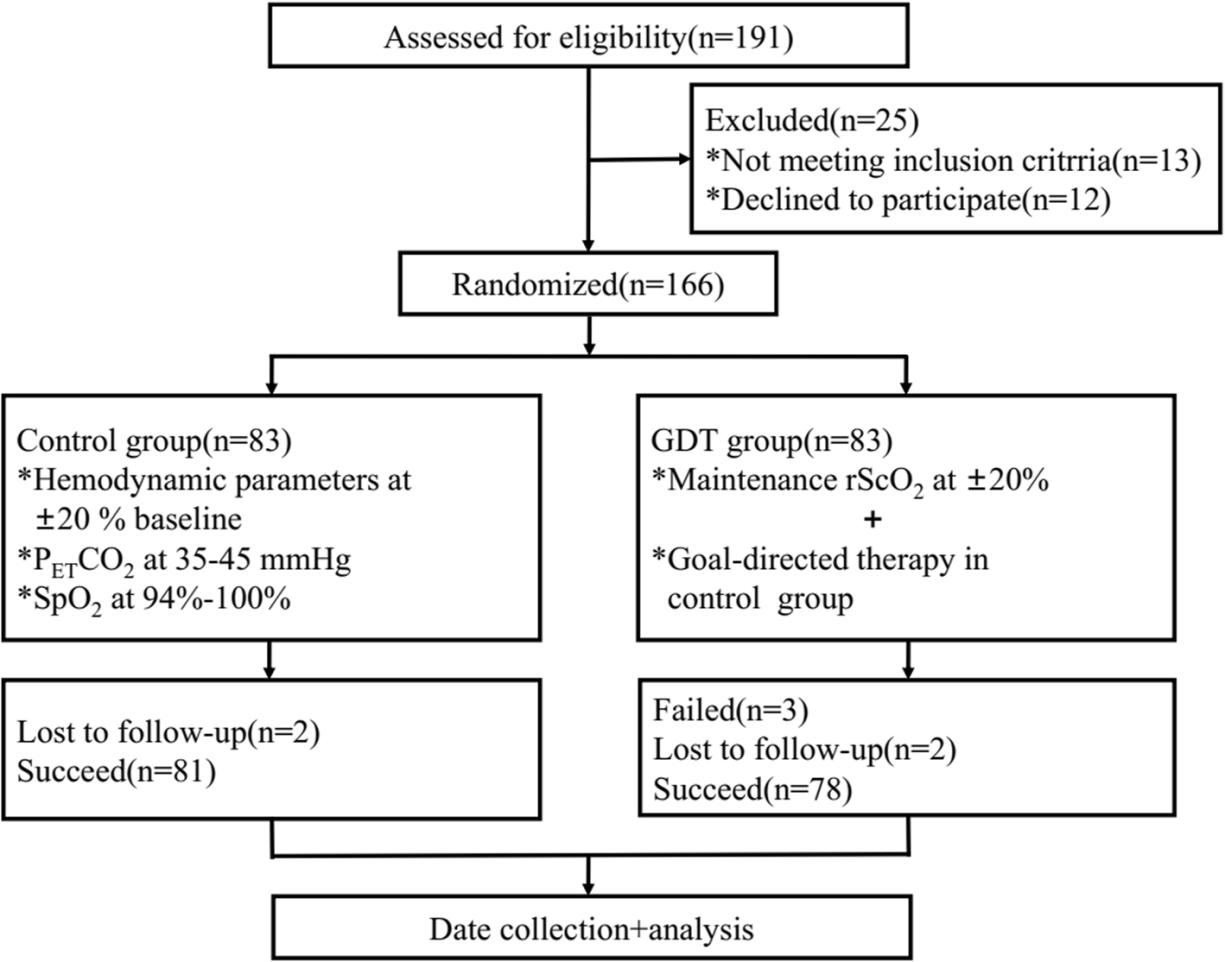
Flow diagram

One hundred and ninety-one patients undergoing thoracoscopic lobectomy under general anesthesia in our hospital from May 2019 to April 2021 were screened for eligibility, and 166 patients were enrolled. According to the odd or even numbers in the random number table, the patients were separated into the control group and the goal-directed therapy group (GDT group), with 83 patients in each group. Inclusion criteria are grade I–II American Society of Anesthesiologists (ASA), age between 65 and 85 years old, and OLV time ≥ 90 min. Exclusion criteria are patients with anemia, important organ dysfunction, inflammation or fever, and psychotropic drugs and patients who could not coordinate to finish Mini-Mental State Examination (MMSE) or whose scores were lower than the corresponding minimum score (illiteracy ≤17, primary school level ≤20, secondary school level ≤22, college level ≤23) [14]. Rejection criteria are patients with intraoperative rScO_2_ value >±20% baseline value or <55% for more than 15 min [15], incomplete research data or midway withdrawal, and perioperative severe complications. Finally, 159 patients were included in this study (Fig. 1).

### Anesthetic methods

Mean artery pressure (MAP), end-tidal carbon dioxide pressure (P_ET_CO_2_), central venous pressure (CVP), percutaneous arterial oxygen saturation (SpO_2_), and heart rate (HR) of patients were routinely monitored and recorded as baseline values after opening the peripheral vein. Propofol, sufentanil, and rocuronium bromide were intravenously injected sequentially, followed by tracheal intubation. Mechanical ventilation was performed after the threaded tube of anesthesia machine and the trachea cannula were prepared: *V*_T_ =5–8 ml/kg, *F* = 12–16 times/min, FiO_2_ = 0.6, PEEP = 0–5 mmHg (1 mmHg = 0.133 kPa), and I:E = 1:2. Catheterization of radial artery and right internal jugular vein was performed. An indwelling occlusion catheter was inserted and checked by fiberoptic bronchoscopy and bilateral lung auscultation.

### Management strategies

Control group: maintenance of the hemodynamic parameters at ±20% baseline, P_ET_CO_2_ at 35–45 mmHg, and SpO_2_ at 94–100%; GDT group: maintenance of the rScO_2_ at ±20% baseline on the basis of the conventional management in the control group [16, 17].

rScO_2_ was monitored by cerebral oxygen saturation monitor (FORE-SIGHT MC-2030C, CAS, USA) after disinfection of the left and right forehead skin of the patient by alcohol cotton pads. During the monitoring, rScO_2_ signals should cover a minimum of two to three grids; otherwise, the position or scaling of electrodes should be reconfirmed. In this study, the mean value of rScO_2_ within 5 min before the beginning of OLV was set as the baseline value, and the mean value of left and right sides was taken for subsequent analysis. When intraoperative rScO_2_ decreased >20% rScO_2_ baseline value or <55% in the GDT group, the following steps were taken to adjust the value until target: the head position was adjusted to avoid the obstruction of venous return in the neck; the position of bronchial occlusion catheter was checked to keep the airway open; the inhaled oxygen concentration was increased to alleviate hypoxemia; the respiratory parameters were adjusted to maintain slightly higher than normal P_ET_CO_2_; the MAP was increased by moderate fluid infusion; infusion of dobutamine (1.5 μg/kg/min) via an intravenous pump was performed to directly increase rScO_2_ [17, 18].

### Observational index

Indexes including patient age, gender, body mass index (BMI), preoperative hemoglobin (Hb) concentration, years of education, preoperative MMSE score, OLV time, hypertension and diabetes cases, lactated Ringer’s solution volume, blood loss, awakening time, cases of agitation during awakening, and postoperative hospital stays were observed and recorded. The levels of interleukin-1β (IL-1β), interleukin-6 (IL-6), tumor necrosis factor-α (TNF-α), and lactate (Lac) concentration were measured before anesthesia (T1), at the end of operation (T2), on day 1 after operation (T3), on day 3 after operation (T4), and on day 7 after operation or before discharge (T5). During T3 to T5, the visual analogue score (VAS) and the incidence of POD in both groups were evaluated and recorded.

### Evaluation and diagnosis of agitation during awakening, intensity of postoperative pain, and POD

The occurrence of agitation during awakening in 15 min after extubation was evaluated with the Riker Sedation-Agitation Scale by specialized personnel, and agitation was defined when the score ≥5 [19]. During T3 to T5, the intensity of postoperative pain and POD was separately evaluated through visual analogue scale (VAS) and confusion assessment method (CAM): CAM score <19 indicates no delirium; CAM score between 20 and 22 indicates suspicious delirium; CAM score > 22 indicates delirium [8, 9].

### ELISA

Five milliliters of peripheral venous blood was extracted from patients and transferred to an anticoagulant tube. A centrifugation was operated at 4000 r/min for 10 min, after which the supernatant was taken out into an EP tube and stored in a refrigerator at −80 °C. Corresponding assay kits for IL-1β (EK0389), IL-6 (EK0410), TNF-α (EK0569), CRP (ZN2120), S100-β (SNM261), and Lac (EK1201) from Boster Biological Technology Corp (Wuhan, China) were applied.

### Sample size estimation

Given the 20% incidence of POD in patients with OLV reported [20] and that the incidence of POD could be reduced to about 5% by goal-directed rScO_2_ monitoring in our previous pre-test, the estimated dropout rate was 5% by taking *α* = 0.05 and *β* = 0.2, and the sample size was at least 154 cases calculated by PASS (version 15.0).

### Statistical analysis

Data were expressed in the mean ± standard deviation (SD), and the POD incidence was represented by a percentage (%). Two types of independent sample *t* test were used for intra-group or inter-group comparisons. A chi-square test was applied to compare the counting data. The repeated measurement analysis of variance (ANOVA) was used for comparisons among different time points, followed by the least significant difference (LSD) method for pairwise comparisons among groups. SPSS (version 24.0) and GraphPad Prism (version 8.0) completed all data analyses of the study.

## Results

### Participant characteristics

Considering the success or failure of goal-directed therapy, the dropout and exclusion of cases, and the compliance of patients, 166 patients were finally enrolled to ensure that the effective sample size was not less than the minimum sample size estimated by PASS and more reliable results, of which 4 patients were excluded due to the incomplete data and 3 patients were excluded for the failure of goal-directed therapy based on rScO_2_. Accordingly, there were 159 cases included in the study, including 81 cases in the control group and 78 cases in the GDT group. There was no statistical difference in the baseline information between the two groups (*P* > 0.05) (Table 1).

**Table 1:** Baseline data of the two groups

Item	Control group (*n* = 81)	GDT group (*n* = 78)	*t/χ*^2^	*P* value
Age (years)	74.1±6.5	72.3±6.6	1.769	0.07
Male [*n* (%)]	35 (43.2)	40 (51.2)	1.039	0.308
BMI (kg/m^2^)	22.8±3.2	23.5±3.3	−1.394	0.165
Hb (g/L)	123.5±14.6	126.9±12.2	−1.593	0.113
Education level (years)	4.7±3.2	5.7±3.2	1.858	0.065
MMSE score	14.6±8.3	16.3±7.3	1.362	0.174
OLV time (min)	110.0±12.7	106.4±14.6	1.655	0.099
Hypertension [*n* (%)]	50 (61.7)	45 (57.6)	0.269	0.603
Diabetes [*n* (%)]	36 (44.4)	24 (30.7)	3.162	0.075
Lactated Ringer’s solution (ml)	1249.6±552.9	1384.7±513.8	−1.593	0.113
Blood loss (ml)	265.0±137.8	249.7±118.5	0.749	0.454

*BMI* body mass index, *Hb* hemoglobin, *MMSE* Mini-Mental State Examination, *OLV* one-lung ventilation

**Fig. 2:**
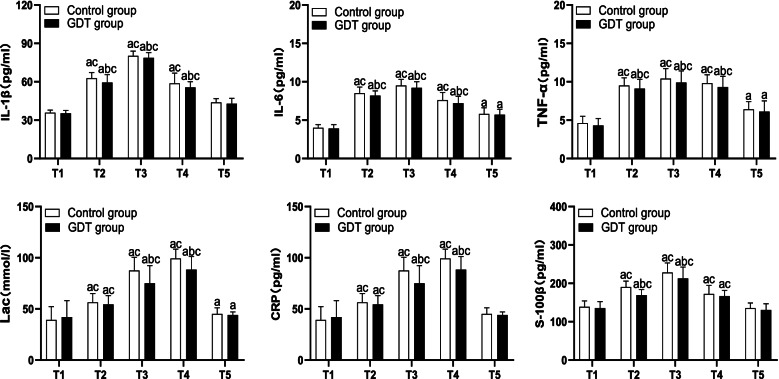
Inflammatory factor, Lac, and S-100β of the two groups. IL, interleukin; TNF-α, tumor necrosis factor-α; Lac, lactate; CRP, C-reactive protein; a refers to *P* < 0.05 as compared to T1; b refers to *P* < 0.05 as compared to the control group; c refers to *P* < 0.05 as compared to T5

The levels of IL-1β, IL-6, TNF-α, Lac, CRP, and S-100β in both two groups during T2 to T4 were higher than those at T1 and T5 (*P* < 0.05), while there was no significant difference at T1 versus T5 (*P* > 0.05). During T2 to T4, the levels of IL-1β, IL-6, TNF-α, and Lac in the control group were higher than those in the GDT group (*P* < 0.05). During T3 to T4, the levels of CRP and Lac in the control group were higher than those in the GDT group (*P* < 0.05). During T2 to T3, the levels of S-100β in the control group were higher than those in the GDT group (*P* < 0.05) (Fig. 2).

The awakening time in the GDT group was earlier than that in the control group, and the POD at T3 were much lower (*P* < 0.05). There was no significant difference in other variables (*P* < 0.05) (Table 2).

**Table 2:** Postoperative recovery of the two groups

Item	Control group (*n* = 81)	GDT group (*n* = 78)	*t/χ*^2^	*P* value
Awakening time (min)	41.5±10.8	38.1±10.5	2.022	0.044
Agitation during awakening [*n* (%)]	14 (17.3)	6 (7.7)	3.324	0.068
Postoperative hospital stay (d)	4.3±1.1	4.6±1.7	−1.167	0.244
VAS score at T3	3.7±1.2	3.6±1.3	0.177	0.859
VAS score at T4	2.1±0.7	1.9±0.8	1.498	0.136
VAS score at T5	1.9±0.8	1.8±0.8	0.578	0.563
Delirium at T3 [*n* (%)]	18 (22.2)	4 (5.1)	9.739	0.001
Delirium at T4 [*n* (%)]	8 (9.8)	3 (3.8)	2.243	0.134
Delirium at T5 [*n* (%)]	2 (2.4)	1 (1.2)	0.302	1.000

## Discussion

The risk of POD mainly has a close correlation with the impairment of cerebral oxygen supply/demand balance during operation. Although the weight of the brain only accounts for 2% of the whole body, the oxygen consumption accounts for 20% [21]. Traditional transcutaneous oxygen saturation monitoring can only reflect the blood oxygen concentration in the whole body of the patient but fail to reflect the real-time blood oxygen utilization of brain tissue, which is an organ with high oxygen consumption. Although the body preferentially transmits blood oxygen to the brain under stress such as surgery, it is still unknown whether cerebral oxygen supply/demand is balanced in the end. In this context, a multi-mode and accurate perioperative anesthetic management strategy based on goal-directed rScO_2_ monitoring emerges, which can provide a favorable supplement to the previous single management mode. The results of the present study indicated that the GDT group was superior to the control group in reduction of inflammatory factors and POD incidence and improvement in tissue perfusion.

Inflammatory factor infiltration is a hot issue in the mechanism of POD. Non-physiological interventions such as surgery and OLV can trigger the increased release of inflammatory factors by decreasing tissue oxygen supply in elderly patients, which in turn brings damages to hippocampal neurons and neurocognitive function through the fragile blood-brain barrier, and the death of neurons further contributes to exacerbation of inflammatory reaction [7]. IL-1β is one of the early warning markers of POD, which affects long-term potentiation by inhibiting the plasticity of hippocampal neurons and synapses to result in compromised memory consolidation [6]. IL-6 and TNF-α are major pro-inflammatory factors in the inflammatory response and play an important role in the development of POD caused by OLV. IL-6 is a reactive protein in the acute stage of inflammation caused by surgery and OLV. After entering the nervous system through the blood-brain barrier, IL-6 can activate microglial cells, astrocytes, and lymphocytes to further aggravate inflammatory response or to modulate the release of neurotransmitters in the endocrine system to inhibit the recovery of cognitive function [22]. TNF-α directly leads to neurodegeneration and synaptic inhibition by revitalizing caspase to activate the death signaling pathway [19]. CRP is highly sensitive to inflammatory factors, and it increases sharply in the acute phase of inflammatory reaction and tissue injury [9]. The S-l00β protein, which is localized to vascular intimal cells, adipose tissue, and muscle tissue, is one of the biochemical indicators that predict and diagnose POD. Single surgical operation is therefore recommended to avoid the influence of different surgical sites on the level of S-l00β [13]. In this study, the results showed that there was no difference in preoperative cognitive function, inflammatory factor levels, and operation time between the two groups. In the GDT group, the imbalance of brain oxygen supply/demand caused by surgery and OLV was improved by taking the rScO_2_ monitoring-based goal-directed therapy, accompanied by inhibited cerebral and peripheral inflammatory reaction, and the rapid recovery of postoperative cognitive function, which reduced the incidence of POD.

Maintaining good tissue perfusion and metabolism is one of the important measures to improve POD. Effective fluid therapy can stabilize and increase cerebral perfusion pressure while also reducing the damage to brain tissue from hypoxia, thereby reducing the activation of glial cell in the brain and inhibiting the release of inflammatory factor to reduce the occurrence of POD [23]. Lactic acid is a metabolite of cells in anaerobic or hypoxic state, and its value directly reflects the condition of systemic tissue perfusion and microcirculation at this stage [24]. The brain is also an indirect indicator that reflects whether the systemic tissue perfusion is perfect. By monitoring the changes of brain oxygen supply/demand in real time, it can reflect the oxygenation and perfusion of other organs and tissues [25]. In elderly patients with OLV, in case that the brain oxygen supply/demand is balanced, the body will deliver oxygen-rich blood to other organs and tissues to reduce the formation of oxygen debt and lactic acid and avoid the release of inflammatory factors due to inadequate tissue perfusion. The application of cerebral oxygen saturation monitoring to guide fluid resuscitation can reduce the demand for infusion, improve systemic perfusion, and reduce the release of inflammatory mediators [25]. In the present study, there was no difference between the two groups in preoperative Lac level and intraoperative infusion of lactate Ringer’s solution. After treatment by different strategies, the Lac in the GDT group was decreased, significantly lower than that in the control group, which suggested that goal-directed rScO_2_ monitoring is beneficial to improve tissue perfusion in elderly patients with OLV, while favorable perfusion and oxygenation can also inhibit the release of inflammatory factors to reduce the occurrence of POD.

Drawbacks: Firstly, this study is single-blinded, which may lead to result bias due to the researchers themselves. Secondly, although there was no significant difference in education level and diabetes between the two groups, the difference was very close to significant. When the two factors overlap, it could cause problems in analysis. Thirdly, different stages of the tumor may also mediate different levels of inflammation. Fourthly, cerebrospinal fluid (CSF) is currently the most efficient body fluid to reflect the severity of cerebral inflammation [26]. However, it is quite difficult to obtain in clinic. Additionally, POD in elderly patients can result in the flow of cerebral inflammatory factors into peripheral blood through fragile blood-brain barrier. The severity of inflammatory reaction in the brain can be indirectly evaluated by measuring the level of inflammatory factors in peripheral blood [27]. Finally, the brain is the organ that the body preferentially supplies oxygen to, and the improvement of the cerebral oxygen supply/demand balance cannot completely exclude hypoxic damages to other organs. In view of the current limitations of this study, the later studies will evaluate which step in GDT is the most important for the improvement of intracranial inflammation and postoperative cognition.

## Conclusion

The rScO_2_ monitoring-based goal-directed therapy can reduce perioperative levels of inflammatory factors, decrease POD incidence, shorten postoperative awakening time, and improve tissue perfusion in elderly patients with OLV.

## Supplementary Information

**Additional file 1:** Riker Sedation-Agitation Scale

## Data Availability

All data generated or analyzed during this study are included in this article and its supplementary information files.

## Abbreviations

ASA: American Society of Anesthesiologists
BMI: Body mass index
CAM: Confusion assessment method
CRP: C-reactive protein
CVP: Central venous pressure
GDT: Goal-directed therapy
Hb: Hemoglobin
IL: Interleukin
OLV: One-lung ventilation
MAP: Mean artery pressure
MMSE: Mini-Mental State Examination
P_ET_CO_2_: End-tidal carbon dioxide pressure
POD: Postoperative delirium
rScO_2_: Regional saturation of cerebral oxygenation
SpO_2_: Oxygen saturation measured by pulse oximetry
TNF-α: Tumor necrosis factor-α
VAS: Visual analogue score
