# Supplementary material for: Goal-directed therapy based on rScO2 monitoring in elderly patients with one-lung ventilation: a randomized trial on perioperative inflammation and postoperative delirium
Source: Trials. 2022 Aug 19;23:687. doi: 10.1186/s13063-022-06654-6 (PMC9389685; doi:10.1186/s13063-022-06654-6)
Supplement: Supplementary file 1 — Additional file 1: Riker Sedation-Agitation Scale [file 13063_2022_6654_MOESM1_ESM.docx]

Riker Sedation-Agitation Scale

| Score | Definition | Description |
| --- | --- | --- |
| 7 | Dangerous agitation | pulling on tracheal tube; trying to pull out various tubes; assaulting medical staff; struggling in bed |
| 6 | Very agitation | requires protective restraint and repeated verbal dissuasion; biting of tracheal tube |
| 5 | Agitation | anxious or physically restless; quiet by verbal dissuasion |
| 4 | Quiet and cooperation | be quiet; wake up easily; obey instructions |
| 3 | Sedation | drowsiness; wakes up with verbal stimulation or gentle shaking and obeys simple commands, but falls asleep quickly |
| 2 | Very sedation | responds to physical stimuli; cannot communicate or obey commands; has voluntary movements |
| 1 | Unable to wake up | no or slight response to malignant stimuli; inability to communicate or obey instructions |
